# Supplementary material for: Vtc4 Promotes the Entry of Phagophores into Vacuoles in the Saccharomyces cerevisiae Snf7 Mutant Cell
Source: J Fungi (Basel). 2023 Oct 11;9(10):1003. doi: 10.3390/jof9101003 (PMC10607680; doi:10.3390/jof9101003)
Supplement: Supplementary file 1 [file jof-09-01003-s001.zip › jof-2579961-supplementary.pdf]

Journal of Fungi

Article

Vtc4 Promotes the Entry of Phagophores into Vacuoles in the *Saccharomyces cerevisiae* Snf7 Mutant Cell

Xiaofan Chen and Yongheng Liang *

Key Laboratory of Agricultural Environmental Microbiology of Ministry of Agriculture, College of Life Sciences, Nanjing Agricultural University, Nanjing 210095, China; 10319117@njau.edu.cn
* Correspondence: liangyh@njau.edu.cn

Supplemental Figures and Legends

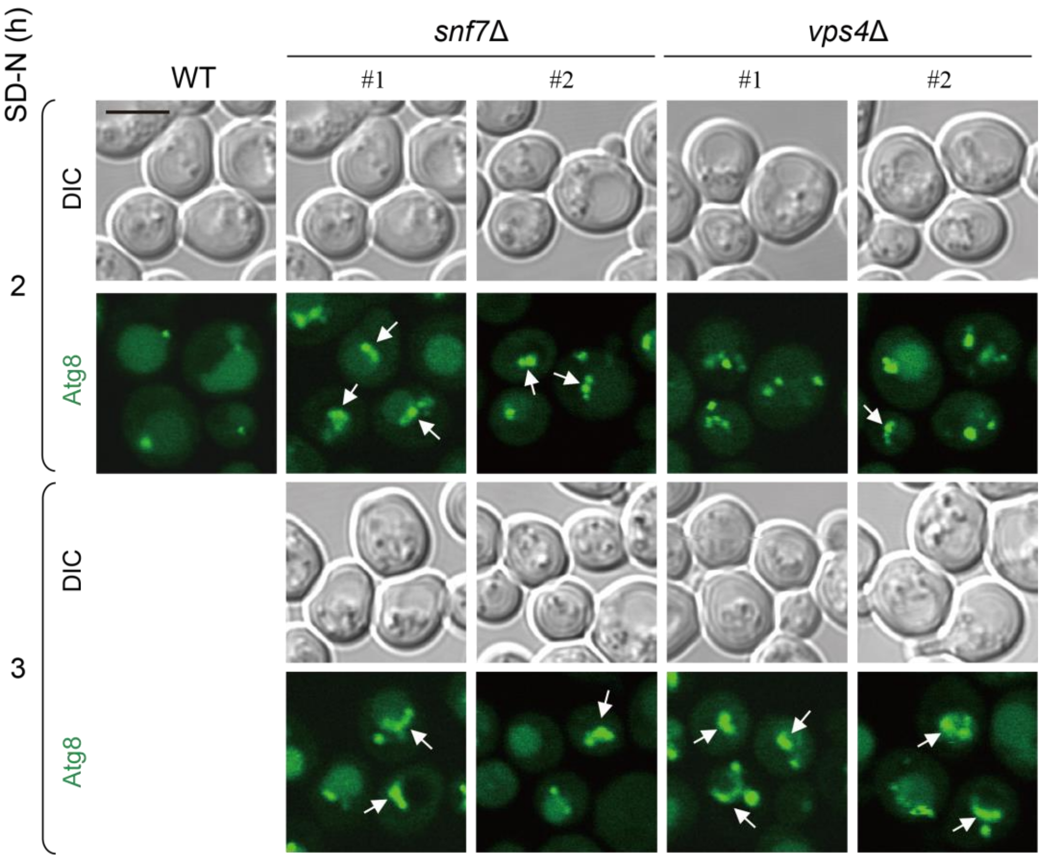

Figure S1. GFP-Atg8 accumulates as clusters in *snf7*Δ and *vps4*Δ cells at 2-3 h of nitrogen starvation. GFP-Atg8 tagged WT, *snf7*Δ and *vps4*Δ cells were grown to mid-log phase in YPD and shifted to SD-N medium for 2 or 3 h, DIC and GFP fluorescence were observed with Leica X confocal microscope. Scales bars at the left-top picture, 5 µm; arrows, phagophore clusters; DIC, differential interference contrast. The results shown represent two independent colonies for each mutant strain. The WT strain starved in SD-N for 2 h was used to show the vacuolar localization of GFP-Atg8 with occasional single dot on vacuolar membrane, indicating a normal autophagy, which is quite different from the accumulated GFP-Atg8 clusters on vacuolar membranes in ESCRT mutant cells.

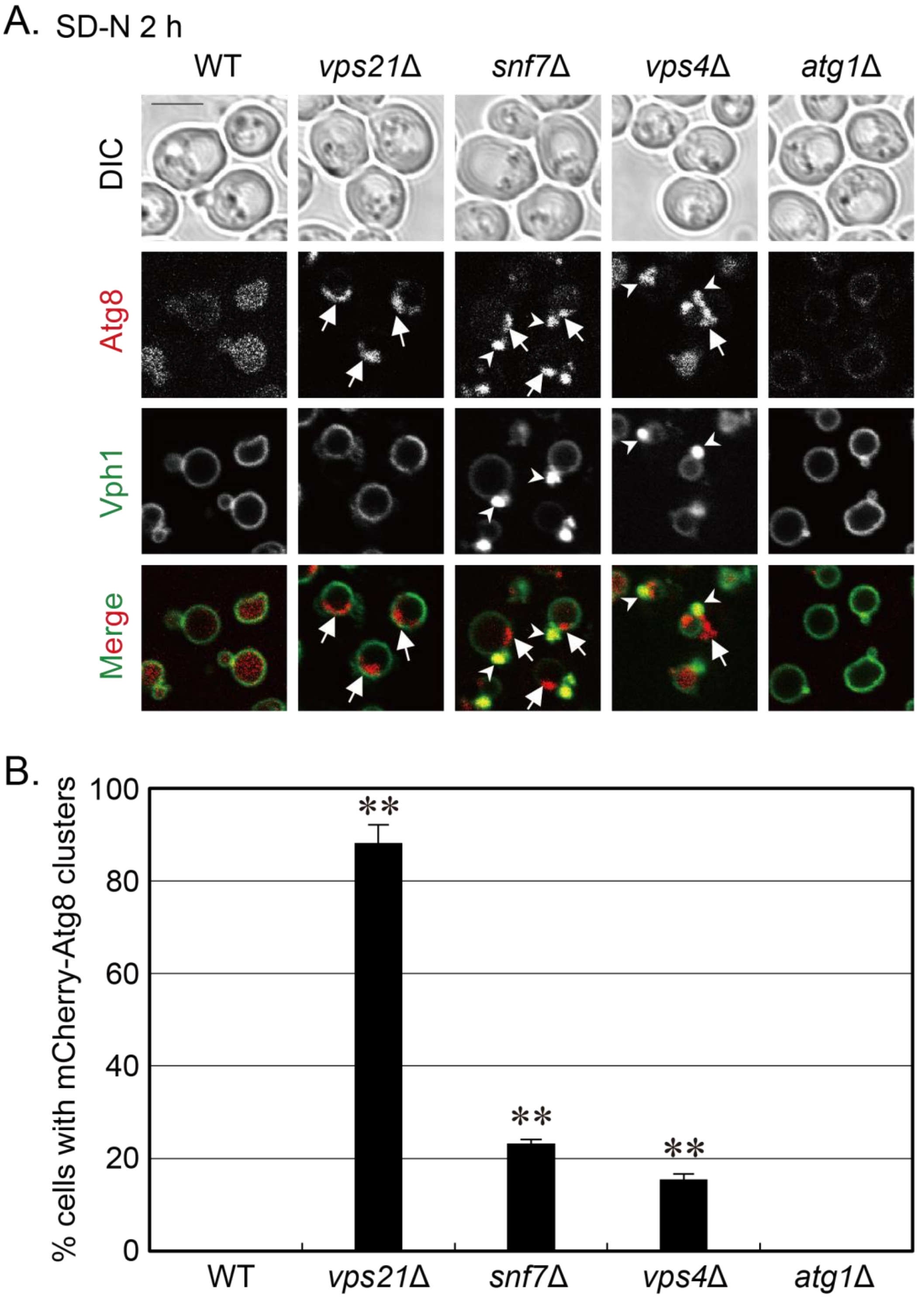

Figure S2. Phagophores labeled with mCherry-Atg8 accumulate on Vph1-GFP-labeled vacuolar membranes in *vps21*Δ and *snf7*Δ cells at 2 h of nitrogen starvation. Indicated cells tagged with mCherry-Atg8 and Vph1-GFP were grown, examined, and presented as in Figure 3. (**A**) The localizations of mCherry-Atg8 and Vph1-GFP in indicated strains. Scales bars at the left-top picture, 5 μm; arrows, mCherry-Atg8 clusters; arrowheads, class E structures; DIC, differential interference contrast. (**B**) Quantification of the percentages of cells with mCherry-Atg8 clusters in the strains represented in panel A. Over 200 cells were counted for each strain. **, $p < 0.01$. The results shown represent two independent experiments.

Supplemental Tables

Table S1. The *S. cerevisiae* yeast strains used in this study.

Strain number	Genotype	Source	Strain name in Figures
SEY6210	*MATα ura3-52 leu2-3,112 his3-Δ200 trp1-Δ901 lys2-801 suc2-Δ9 GAL*	[26]	Parent strain
YLY2422	SEY6210, *ura3::GFP-ATG8-URA3*	[9]	WT (wild-type) in Fig. 1, 2
YLY1628	YLY2422, *vps21::kanMX3*	[15]	*vps21Δ* in Fig. 1, 2
YLY1848	YLY2422, *vps21::LYS2 vtc4::kanMX3*	[15]	*vps21Δvtc4Δ* in Fig. 1, 2
YLY6918	YLY2422, *snf7::hphMX4*	[15]	*snf7Δ* in Fig. S1
YLY6405	YLY2422, *snf7::kanMX3*	[15]	*snf7Δ* in Fig. 1, 2
YLY12201	YLY6918, *vtc4::kanMX3*	This study	*snf7Δvtc4Δ* in Fig. 1, 2
YLY6735	YLY2422, *vps4::hphMX4*	[15]	*vps4Δ* in Fig. S1
YLY1843	YLY2422, *vtc4::kanMX3*	[15]	*vtc4Δ* in Fig. 1, 2
YLY5928	YLY2422, *atg1::kanMX3*	[9]	*atg1Δ* in Fig. 1, 2
YLY1735	SEY6210, *VPH1-GFP::HIS3MX6 ATG8::mCherry-ATG8-TRP1*	[15]	WT in Fig. 3, S2
YLY1736	YLY1735, *vps21::hphMX4*	[15]	*vps21Δ* in Fig. 3, S2
YLY12203	YLY1736, *vtc4::kanMX3*	This study	*vps21Δvtc4Δ* in Fig. 3
YLY1737	YLY1735, *snf7::hphMX4*	This study	*snf7Δ* in Fig. 3, S2
YLY12204	YLY1737, *vtc4::kanMX3*	This study	*snf7Δvtc4Δ* in Fig. 3
YLY1738	YLY1735, *vps4::hphMX4*	This study	*vps4Δ* in Fig. S2
YLY12202	YLY1735, *vtc4::kanMX3*	This study	*vtc4Δ* in Fig. 3
YLY1741	YLY1735, *atg1::kanMX3*	This study	*atg1Δ* in Fig. 3, S2

Table S2. Primers used for tagging or deletion in this study.

Primer number	Primer name	Sequence
YLO-2756	*VTC4*+1000-For	TATGGTCCACCTTTACTCCA
YLO-2757	*VTC4*+464-Rev	CGTTGTTCAGCCACTTTATG
YLO-877	Ptef	ACCCATGGTTGTTTATGTTC
YLO-754	*SNF7*+500-For	CGCATCAAAGAAAGAGGTAG
YLO-755	*SNF7*+500-Rev	CAGGGCGAAGTAATCCAAAG
YLO-741	*VPS4*+550-For	GAGCGAGACAACCTCAAACC
YLO-742	*VPS4*+500-Rev	AGGAAAGCATCTCTGGGACT
YLO-2623	*ATG1*+500-For	TTCTTTTTAACCGCTCGGCT
YLO-2624	*ATG1*+500-Rev	GGATATGTATAGCCAAAGGC
